# Clinical characteristics and Co-morbidities among patients admitted with COVID-19

**DOI:** 10.1016/j.amsu.2022.103898

**Published:** 2022-05-31

**Authors:** Omair Al Hussain

**Affiliations:** Head of Otolaryngology and Head & Neck Surgery Department, College of Medicine, Imam Mohammad Ibn Saud Islamic University (IMSIU), Riyadh, Saudi Arabia

**Keywords:** Comorbidities, Pneumonia, Acute respiratory disorder syndrome, COVID-19

## Abstract

**Background:**

During the Coronavirus Disease-2019 (COVID-19) pandemic, several characteristics of COVID-19 patients, based on demographics, clinical symptoms, and the presence of comorbidities, were found to be associated with the complications developed. COVID-19 symptoms vary greatly and are more prominent with comorbid diseases. Therefore, the aim of this study to find the clinical characteristics and its association with different comorbidities.

**Methods:**

This is a retrospective study that was performed on the data obtained from medical records of 3999 patients in Riyadh. Demographic data, clinical symptoms and comorbidities were noted on the day of hospital admission. Complications developed during the COVID -19 infection were observed.

**Results:**

The average age of patients were 49.55 years old. Fever was the most common symptom among the patients (85.85%), followed by cough (85.85%), and shortness of breath (83.25%). The most common comorbidities were diabetes mellitus (39.51%), hypertension (33.91%), and asthma (9.45%), with chronic rhinosinusitis being the least common (0.5%). Pneumonia affected 61.90% of the patients admitted to the hospital. Furthermore, 8.73% got acute respiratory distress syndrome (ARDS), and 7.25% acquired pneumonia and Acute ARDS simultaneously. Co-morbidities were significantly correlated with complications developed during COVID-19.

**Conclusion:**

Hypertension and diabetes mellitus were two of the most common symptoms observed. Clinical symptoms, comorbidities, and complications are higher in female patients compared to male patients and most of the patients’ developed complications.

## Highlights

• Longitudinal data of hospitalized COVID-19 patient is beneficial for future pandemics.
• Comorbidities is key factor in any kind of outbreaks, in our study, females were at more risk of developing complications.
• Comorbidities can differ regarding rates of hospitalization and infection.

## 1 Introduction

Coronavirus disease 2019 (COVID-19), caused by a highly contagious virus known as severe acute respiratory syndrome coronavirus 2 (SARS-CoV-2), first appeared in China around the end of 2019 and quickly spread across the globe. The World Health Organization declared COVID-19 a pandemic in March 2020. Experts in the field of medicine are still discovering more about the natural course and severity of COVID-19. Previous studies have explained the baseline characteristics, symptoms, and clinical outcomes of patients with COVID-19 [1].

COVID-19 symptoms vary greatly, ranging from asymptomatic to mild to severe pneumonia-like symptoms [2]. ARDS is caused by a large number of COVID-19 pneumonia cases, and it usually develops on day eight or nine after the onset of symptoms [3]. A substantial number of COVID-19 pneumonia patients admitted to the ICU have comorbidities that have a detrimental impact on the disease's prognosis [4]. Several studies have already described clinical features, demographics, and laboratory data parameters at the time of admission. Moreover, a number of studies have detailed the clinical outcomes, comorbidities, and complications [5]. However, the fact that many patients were still in hospitals at the time of publication skewed the results. As a result, more reliable data on co-morbidities and complications is needed. Furthermore, a large number of samples is required to better understand the association of comorbidities of COVID-19 patients with complications developed during the disease.

This study describes the baseline characteristics, symptoms, comorbidities, and complications developed during COVID-19 patients admitted to Prince Mohammed Bin Abdulaziz Hospital in Riyadh.

## 2 Methodology

### 2.1 Patients and study design

This is a retrospective descriptive study that was conducted in patients admitted with COVID-19 infection between March 2020 to April 2021 in Prince Mohammed Bin Abdulaziz Hospital in Riyadh. The study included all the confirmed cases of COVID-19 Patients. The primary objective of this study was to investigate clinical characteristics, symptoms and comorbidities of COVID-19 patients.

All of the information was gathered from hospital medical records and included demographic information, clinical symptoms and signs, and complications that developed during the COVID-19 disease. Observed comorbidities were Diabetes Mellitus (DM), Hypertension (HTN), asthma, and Chronic Rhinosinusitis (CRS). On the day of admission to the hospital, the following clinical symptoms were noted: fever, cough, shortness of breath, diarrhoea, fatigue, vomiting, headache, sore throat, nausea, loss of appetite, malaise, chest pain, abdominal pain, loss of smell, loss of taste, dizziness, runny nose, rhinorrhoea, and palpitation. Most of the patients experienced complications such as pneumonia, Acute Respiratory Distress Syndrome (ARDS), and septic shock during COVID-19. The research has been registered with research registry with registration number: researchregistry7821. In addition, this article has been submitted in line with the STROCSS guidelines [6].

### 2.2 Statistical analysis

The data was entered into Microsoft Excel, which cleaned it all up for analysis. Variables were then imported into IBM SPSS (Statistical Package for Social Sciences) version 26 for statistical analysis. Descriptive analysis of baseline and clinical characteristics of patients was performed. Qualitative variables were expressed as numbers and percentages, while quantitative variables were expressed as means, medians, and interquartile ranges (IQR). The Pearson correlation coefficient was used to examine the relationship between the number of comorbidities and complications developed during the COVID-19 infection, and the chi-square test was used to examine the association. A p-value of 0.05 was considered statistically significant.

## 3 Results

Data was collected from patients admitted to the hospital between March 2020 and April 2021 from a total of 3999 COVID-19 patients. The average age and standard deviation of the male patients, was 51.59 ± 16.12 and female was 48.83 ± 14.15. The median age of male patients was 53 and the median age for female patients was 48. The mean value of the Body Mass Index (BMI) of patients was 29.48 ± 6.94. It has been observed that asymptomatic COVID-19 cases were higher in male patients (67.5%) compared to female patients (32.5%). Among 3999 cases, 1353 (33.83%) cases were from Saudi Arabia, 10.50% from India and 7.68% from Syria (Table 1).

**Table 1:** Baseline characteristics of patients admitted with COVID-19.

	Descriptive statistics	
Age		
MEAN ± SD	49.55 ± 14.75	
Median (IQR)	49 [22]	
BMI		
MEAN ± SD	29.48 ± 6.94	
Median (IQR)	28 [8]	
	Frequency (n)	Percentage (%)
Gender		
Female	1049	26.23
Male	2950	73.77
Asymptomatic	154	3.85
Symptomatic	3845	96.15
Nationality		
Afghan	13.0	0.33
Bangladesh	195.0	4.88
British	4.0	0.10
Canada	4.0	0.10
Egyptian	322.0	8.05
Eriterian	19.0	0.48
Ethiopian	7.0	0.18
Filipino	271.0	6.78
Indian	420.0	10.50
Indonesia	137.0	3.43
Iraq	5.0	0.13
Jordan	80.0	2.00
Lebanon	18.0	0.45
Morocco	11.0	0.28
Nepal	26.0	0.65
Pakistan	177.0	4.43
Palestine	68.0	1.70
Philippine	31.0	0.78
Saudi	1353.0	33.83
Sri lanka	19.0	0.48
Sudan	148.0	3.70
Syrian	307.0	7.68
Yemen	241.0	6.03
Others	123.0	3.08

IQR, Inter Quartile Range; SD, Standard Deviation; BMI; Body Mass Index.

Fever was the most common prevalent symptom (85.85%) followed by cough (85.85%), shortness of breath (83.25%), diarrhoea (17.43%), fatigue (16.2%), vomiting (15.38%), headache (15.23%), sore throat (9.3%) and nausea (8.5%). Loss of appetite (8%), malaise (6.35%), chest pain (7.28%), abdominal pain (4.78%), loss of smell (2.5%), loss of taste (2.4%), dizziness (2%), runny nose (1.08%), rhinorrhoea (0.28%), and palpitation (0.28%) were among the other least common symptoms found. Symptoms such as fever (80.7%), cough (86.5%), and shortness of breath (84%) were higher in male patients compared to female patients (Table 2).

**Table 2:** Clinical characteristics of patients admitted with COVID-19.

Symptoms	Frequency (n)	Percentage (%)
Fever	3433	85.85
Cough	3433	85.85
Shortness of breath	3329	83.25
Diarrhoea	697	17.43
Fatigue	648	16.2
Vomiting	615	15.38
Headache	609	15.23
Sore throat	372	9.3
Nausea	340	8.5
Loss of appetite	320	8
Malaise	254	6.35
Chest pain	291	7.28
Abdominal pain	191	4.78
Loss of smell	100	2.5
Loss of taste	96	2.4
Dizziness	80	2
Runny nose	43	1.08
Rhinorrhoea	11	0.28
Palpitation	11	0.28

**Fig. 1:**
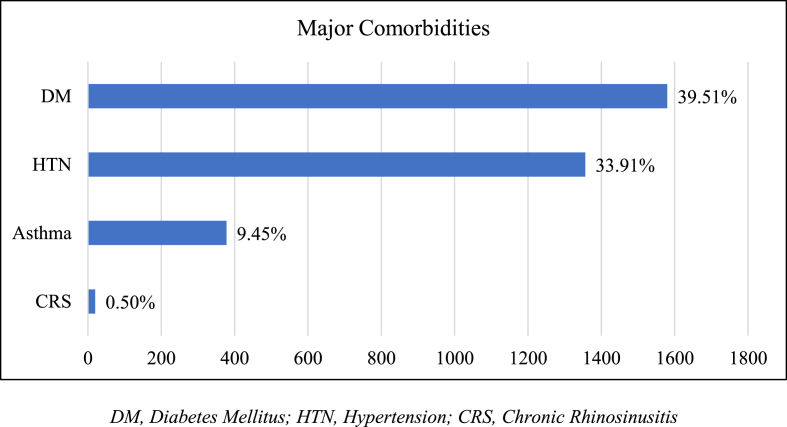
Comorbidities of patients admitted with COVID-19. DM, Diabetes Mellitus; HTN, Hypertension; CRS, Chronic Rhinosinusitis.

The most common comorbidity was DM (39.51%), followed by hypertension (33.91%), and asthma (9.45%) among the 3999 COVID-19 patients, while CRS was the least common comorbidity (0.50%) (Fig. 1). Furthermore, 61.90% had pneumonia, 8.73% had ARDS, and 7.25% developed pneumonia and ARDS at the same time. Lastly, Septic shock affected 0.4% of the total patients (Table 3).

**Table 3:** Developed condition of COVID-19 patients.

Developed Conditions	Frequency (n)	Percentage (%)
Pneumonia	2447	61.19
Acute Respiratory Distress Syndrome (ARDS)	349	8.73
Septic shock	17	0.43
Pneumonia + ARDS	290	7.25

ARDS: Acute Respiratory Distress Syndrome.

The majority of patients were in the age group of 50–59 years, with males accounting for 25.2% and females accounting for 22.6% of the total group. DM was the most common comorbidity among females, accounting for 42.61% of all females, followed by hypertension (42.52%), asthma (12.96%), and CRS (0.76%). In males, DM accounted for 38.54%, followed by Hypertension (30.85%), Asthma (8.20%) and CRS (0.37%) of the total population. Male patients were shown to have a lower prevalence of diabetes (4.07%), hypertension (11.76%), and asthma (4.76%) than female patients (Table 4).

**Table 4:** Characteristics of COVID-19 patients by age, sex, comorbidities and developed conditions.

	Female (n = 1049)	Male (n = 2950)
**Age group**		
<10	4 (0.4)	7 (0.2)
11-18	9 (0.9)	13 (0.4)
19-29	64 (6.1)	184 (6.3)
30 - 39	198 (19)	631 (21.5)
40 - 49	179 (17.2)	708 (24.1)
50 - 59	236 (22.6)	741 (25.2)
60 - 69	219 (21)	417 (14.2)
70 -79	88 (8.4)	179 (6.1)
>80	46 (4.4)	60 (2)
Symptomatic	999 (95.23)	2846 (96.47)
Asymptomatic	50 (4.77)	104 (3.53)
**Comorbidities**		
DM	447 (42.61)	1137 (38.54)
HTN	446 (42.52)	910 (30.85)
Asthma	136 (12.96)	242 (8.20)
CRS	8 (0.76)	11 (0.37)
**Developed conditions**		
Pneumonia	688 (65.59)	1759 (59.63)
ARDS	92 (8.77)	257 (8.71)
Pneumonia + ARDS	87 (8.29)	221 (7.49)
Septic shock	4 (0.38)	13 (0.44)

n: numbers, DM: Diabetes Mellitus; HTN: Hypertension, CRS: Chronic Rhinosinusitis, ARDS: Acute Respiratory Distress Syndrome.

From our study, 701 female patients (66.82%) and 1793 male patients (60.6%) had developed respiratory issues such as pneumonia, ARDS, and septic shock during the COVID-19 period. Female COVID-19 patients were shown to have a higher rate of respiratory problems than male COVID-19 patients. Moreover, 65.59% of the female patients acquired pneumonia, 8.77% had ARDS, 8.29% had both ARDS and pneumonia, and 0.38%had septic shock (Table 4). There was a statistically significant correlation observed between comorbidities and complications developed during COVID-19 disease; 29% of the patients had one co-morbidity, 26.2% of the patients had two co-morbidities, and 15.67% of the patients had more than three comorbidities and developed complications during COVID-19 (Table 5).

**Table 5:** Number of comorbidities and correlation with clinically developed complication.

Number of comorbidities	No (%) of patients	Chis square p value	Pearson correlation p value	Correlation coefficient
1	1160 (29%)	<0.001	<0.001	0.067
2	1047 (26.2%)	<0.001	<0.001	0.130
≥3	627 (15.67%)	<0.001	<0.001	0.102

## 4 Discussion

In this descriptive study, we analysed the baseline characteristics, clinical symptoms, and complications developed during the COVID-19 infection of 3999 patients admitted to Prince Mohammed Bin Abdulaziz Hospital in Riyadh. In our study, the median age of the male patients was higher than that of female patients in most of the patients, both male and female were in the age group of 50–59 years. COVID-19 infection was found to be higher compared to the younger age group. Symptomatic COVID-19 was found to be higher in both sex. Fever and cough were the most commonly reported clinical symptoms in patients with COVID-19 infection [[7], [8], [9], [10]]. In our study, the most prevalent symptom were fever, cough and shortness of breath. A study of approximately 370,000 confirmed COVID-19 cases with known symptom status reported to the Centers for Disease Control and Prevention (CDC) in the United States highlighted the variety of related symptoms similar to our study [11]. Obesity-related comorbidities have been linked to more severe COVID-19 complications and higher mortality, whereas a high BMI has been associated with hospitalization and poor survival [12]. In this study it has been observed that patients had higher BMI (29.48 ± 6.94).

Studies have shown a higher mortality rate in COVID-19 patients with pre-existing comorbidities to the patient without comorbidity. The most often reported comorbidity in COVID-19 patients are hypertension, DM and cardiovascular disease [13,14] We found that, regarding comorbidities such as DM, hypertension was the more common symptom observed in patients at the time of hospital admission. In our study female patients had higher proportion of comorbidities such as hypertension and DM similar to other studies [[15], [16], [17]]. There is a greater fear that patients who have asthma will get more complications and be more likely to get COVID-19. But from our study, it shows that overall 9.45% of the COVID-19 patients had existing asthma problems, of which 12.96% of the patients were females and 8.20% were males. Currently, there is no strong evidence to prove that the increased infection rate of COVID-19 with those asthma conditions. However, its actual contribution to risk may be influenced by the presence of other behavioural factors, such as smoking and those associated with other comorbidities [18]. We found a higher incidence of pneumonia and ARDS during the COVID infection. In a stratified analysis, we observed that female patients developed more complications such as pneumonia and ARDS [[19], [20], [21], [22]].

Wang et al. [23] reported that 46.4% of patients with COVID-19 had multiple comorbidities, and 72.2% of comorbid patients required ICU hospitalization. Another recent meta-analysis reported that severe COVID-19 was highly associated with the underlying diseases such as HTN (21.1%), DM (9.7%), and respiratory system diseases [24]. Co-morbidities were significantly correlated with complications developed during the COVID-19. Twenty nine percent of the patients had at least one major comorbidity. In addition to that, 26.2% of the patients had at least two comorbidities, and 15.67% of the patients had more than three comorbidities. These patients developed clinical complications such as pneumonia, ARDS, and septic shock [25]. In our study, it has been found that there was a significant association between comorbidities and complications developed during COVID -19.

Several studies found severe COVID-19 to be accompanied by comorbidities [18,23,25,26] and also added evidence to that highlighting the number of comorbidities as a strong risk factor for COVID-19 severity [27,28]. A large sample study conducted by Chudasama et al. described that multimorbidity was associated with a greater risk of outcomes in COVID-19 [29]. Our study results are also strongly consistent with previous studies that found the rise in the number of co morbidities increases the risk of complications or outcomes.

The study analysis was conducted with a limited number of variables and those are not sufficient to determine the risk factor more precisely. Lack of information like severity, survival data, length of hospital stay, and ventilator stay might lead to bias. The quality of different studies was different from each other. Different studies analysed different factors to assess the risk factor of comorbidities associated with the severity and clinically developed complications of COVID-19 patients. Secondly, the judgment criteria for severe and non-severe patients were not included in the study. Future studies should evaluate the impact of different comorbidities on COVID-19 severity and mortality using more detailed models.

## 5 Conclusion

This study demonstrated that there is a significant correlation between comorbidities and complications developed during the COVID-19. Clinical symptoms, comorbidities and complications is higher in female patients compared to male patients. In this study, diabetes mellitus and hypertension were the most prevalent comorbidities in patient with COVID -19 infection, and patient with co morbidities developed more complications. Hence, presence of comorbidities would be the risk for developing complications during the COVID-19 infection.

## Ethical approval

The study was approved by the Scientific and Research Ethics Committee of the University of Prince Mohammed Bin Abdulaziz Hospital in Riyadh.

## Funding

None.

## Ethical approval

Research studies involving patients require ethical approval. Please state whether approval has been given, name the relevant ethics committee and the state the reference number for their judgement.

Institutional review board approval was obtained accordingly.

## Guarantor

The Guarantor is the one or more people who accept full responsibility for the work and/or the conduct of the study, had access to the data, and controlled the decision to publish.

## Consent

Informed consent was obtained according and in guidelines of the declaration of Helsinki.

## Author contributions

Please specify the contribution of each author to the paper, e.g. study concept or design, data collection, data analysis or interpretation, writing the paper, others, who have contributed in other ways should be listed as contributors.

All authors contributed evenly to the conceptualization, drafting, data analysis, writing and proofreading of the research.

## Registration of research studies

1. Name of the registry: Research Registry
2. Unique Identifying number or registration ID: researchregistry7821
3. Hyperlink to your specific registration (must be publicly accessible and will be checked): https://www.researchregistry.com/register-now#user-researchregistry/registerresearchdetails/625eb36011413d001f745d9b/

## Declaration of competing interest

The author declares no conflict of interest.

## References

[1] Šabanović Adilović A., Rizvanović N., Kovačević M., Adilović H. (2021). Clinical characteristics, comorbidities and mortality in critically ill mechanically ventilated patients with Covid-19: a retrospective observational study. Med. Glas. : official publication of the Medical Association of Zenica-Doboj Canton, Bosnia and Herzegovina.

[2] Santus P., Radovanovic D., Saderi L., Marino P., Cogliati C., De Filippis G. (2020). Severity of respiratory failure at admission and in-hospital mortality in patients with COVID-19: a prospective observational multicentre study. BMJ Open.

[3] Gibson P.G., Qin L., Puah S.H. (2020). COVID-19 acute respiratory distress syndrome (ARDS): clinical features and differences from typical pre-COVID-19 ARDS. Med. J. Aust..

[4] Zádori N., Váncsa S., Farkas N., Hegyi P., Erőss B. (2020). The negative impact of comorbidities on the disease course of COVID-19. Intensive Care Med..

[5] Hussain M., Iltaf S., Sr, Salman S., Sr, Ghuman F., Abbas S., Fatima M. (2021). Frequency of comorbidities in admitting COVID-19 pneumonia patients in a tertiary care setup: an observational study. Cureus.

[6] Mathew G., Agha R. (2021). Strocss 2021: strengthening the reporting of cohort, cross-sectional and case-control studies in surgery. Int. J. Surg..

[7] Alahmari A.A., Khan A.A., Elganainy A., Almohammadi E.L., Hakawi A.M., Assiri A.M. (2021). Epidemiological and clinical features of COVID-19 patients in Saudi Arabia. Journal of Infection and Public Health.

[8] Liu C.L., Lu Y.T., Peng M.J., Chen P.J., Lin R.L., Wu C.L. (2004). Clinical and laboratory features of severe acute respiratory syndrome vis-a-vis onset of fever. Chest.

[9] Halim A.A., Alsayed B., Embarak S., Yaseen T., Dabbous S. (2016). Clinical characteristics and outcome of ICU admitted MERS corona virus infected patients. Egypt. J. Chest Dis. Tuberc..

[10] Hannawi S., Hannawi H., Naeem K.B., Elemam N.M., Hachim M.Y., Hachim I.Y. (2021). Clinical and laboratory profile of hospitalized symptomatic COVID-19 patients: case series study from the first COVID-19 center in the UAE. Front. Cell. Infect. Microbiol..

[11] Stokes E.K., Zambrano L.D., Anderson K.N., Marder E.P., Raz K.M., El Burai Felix S. (2020). Coronavirus disease 2019 case surveillance - United States, january 22-may 30, 2020. MMWR Morbidity and mortality weekly report.

[12] Albashir A.A.D. (2020). The potential impacts of obesity on COVID-19. Clin. Med..

[13] Harrison S.L., Fazio-Eynullayeva E., Lane D.A., Underhill P., Lip G.Y.H. (2020). Comorbidities associated with mortality in 31,461 adults with COVID-19 in the United States: a federated electronic medical record analysis. PLoS Med..

[14] Du Y., Zhou N., Zha W., Lv Y. (2021). Hypertension is a clinically important risk factor for critical illness and mortality in COVID-19: a meta-analysis. Nutrition, metabolism, and cardiovascular diseases. Nutr. Metabol. Cardiovasc. Dis..

[15] Sanyaolu A., Okorie C., Marinkovic A., Patidar R., Younis K., Desai P. (2020). Comorbidity and its impact on patients with COVID-19. SN Compr Clin Med.

[16] Ng W.H., Tipih T., Makoah N.A., Vermeulen J.G., Goedhals D., Sempa J.B. (2021). Comorbidities in SARS-CoV-2 patients: a systematic review and meta-analysis. mBio.

[17] Bajgain K.T., Badal S., Bajgain B.B., Santana M.J. (2021). Prevalence of comorbidities among individuals with COVID-19: a rapid review of current literature. Am. J. Infect. Control.

[18] Lombardi C., Gani F., Berti A., Comberiati P., Peroni D., Cottini M. (2021). Asthma and COVID-19: a dangerous liaison?. Asthma Research and Practice.

[19] Gibson P.G., Qin L., Puah S.H. (2020). COVID-19 acute respiratory distress syndrome (ARDS): clinical features and differences from typical pre-COVID-19 ARDS. Med. J. Aust..

[20] Attaway A.H., Scheraga R.G., Bhimraj A., Biehl M., Hatipoğlu U. (2021). Severe covid-19 pneumonia: pathogenesis and clinical management. BMJ.

[21] Qiu P., Zhou Y., Wang F., Wang H., Zhang M., Pan X. (2020). Clinical characteristics, laboratory outcome characteristics, comorbidities, and complications of related COVID-19 deceased: a systematic review and meta-analysis. Aging Clin. Exp. Res..

[22] Ejaz H., Alsrhani A., Zafar A., Javed H., Junaid K., Abdalla A.E. (2020). COVID-19 and comorbidities: deleterious impact on infected patients. Journal of Infection and Public Health.

[23] Wang D., Hu B., Hu C., Zhu F., Liu X., Zhang J. (2020). Clinical characteristics of 138 hospitalized patients with 2019 novel coronavirus-infected pneumonia in wuhan, China. JAMA.

[24] Yang J., Zheng Y., Gou X., Pu K., Chen Z., Guo Q. (2020). Prevalence of comorbidities and its effects in patients infected with SARS-CoV-2: a systematic review and meta-analysis. Int. J. Infect. Dis. : IJID : official publication of the International Society for Infectious Diseases.

[25] Hasan M.J., Anam A.M., Huq S.M.R., Rabbani R. (2021). Impact of comorbidities on clinical outcome of patients with COVID-19: evidence from a single-center in Bangladesh. Health Scope.

[26] Alam M.R., Kabir M.R., Reza S. (2021). Comorbidities might be a risk factor for the incidence of COVID-19: evidence from a web-based survey. Preventive Medicine Reports.

[27] Ge E., Li Y., Wu S., Candido E., Wei X. (2021). Association of pre-existing comorbidities with mortality and disease severity among 167,500 individuals with COVID-19 in Canada: a population-based cohort study. PLoS One.

[28] Dessie Z.G., Zewotir T. (2021). Mortality-related risk factors of COVID-19: a systematic review and meta-analysis of 42 studies and 423,117 patients. BMC Infect. Dis..

[29] Chudasama Y.V., Zaccardi F., Gillies C.L., Razieh C., Yates T., Kloecker D.E. (2021). Patterns of multimorbidity and risk of severe SARS-CoV-2 infection: an observational study in the U.K. BMC Infect. Dis..

